# Spatio-Temporal Analysis and Clinical-Epidemiological Characterization of Visceral Leishmaniasis in Maranhão, Brazil, from 2009 to 2020

**DOI:** 10.3390/tropicalmed9040076

**Published:** 2024-04-05

**Authors:** Carolina Azevedo Amaral, Taciana Mirely Maciel Higino, Karen Fernanda Castro Silva, Nathalia Rodrigues dos Reis, Mariana Gomes Pereira, Rita de Cássia Mendonça de Miranda, Amanda Silva dos Santos Aliança

**Affiliations:** 1 Postgraduate Program in Microbial Biology, Programa de Pós-Graduação em Biologia Microbiana, Universidade CEUMA, São Luís 65075-120, MA, Brazil; carolinaraazevedo@gmail.com (C.A.A.); marigomesmed0@gmail.com (M.G.P.); 2 Fundação Altino Ventura, Recife 50070-040, PE, Brazil; taciana.higino@doefav.com; 3 Curso de Graduação em Biomedicina, Universidade CEUMA, São Luís 65075-120, MA, Brazil; karenfernanda414@gmail.com (K.F.C.S.); nathalia.2001.nr56@gmail.com (N.R.d.R.); 4 Programa de Pós-Graduação em Meio Ambiente, Universidade CEUMA, São Luís 65075-120, MA, Brazil; rita.miranda@ceuma.br

**Keywords:** Visceral Leishmaniasis, spatial distribution, epidemiology profile, clinical profile

## Abstract

This study was carried out to identify the spatial distribution and characterize the clinical–epidemiological profile of Visceral Leishmaniasis (VL) in Maranhão state, Brazil, from 2009 to 2020. This descriptive ecological study collected sociodemographic and clinical data of VL cases from the Brazilian Notifiable Diseases Information System database. A spatial autocorrelation analysis (Moran statistics) was performed. From 2009 to 2020, 5699 cases of VL were reported, with incidence of 6.5 cases/100,000 and prevalence of 7.1 cases/100,000. The temporal analysis showed a significant growth in incidence from 2009 to 2018, followed by a significant decrease between 2019 and 2020. The Moran map shows hotspots of high values in the central–west and central–east regions, and hotspots of low values in the northern region of Maranhão. The profile of patients affected by VL comprises males (OR = 1.8; IC95% = 1.72–1.92), aged under 14 years, brown, and with incomplete elementary schooling. The main symptoms reported were fever, fatigue, and edema. The main diagnostic method was laboratory. The mortality rate was 6.8%, and co-infection with HIV was reported by 8.5% of patients. The results of this study indicated the increase in incidence and lethality, as well as the expansion, of leishmaniasis in the state of Maranhão.

## 1. Introduction

Visceral Leishmaniasis (VL), also known as Calazar, cases have been reported in at least 12 Latin American countries, with 96.6% of cases occurring in Brazil [1,2]. VL is a zoonosis caused by intracellular protozoa of the genus *Leishmania*, of which more than 20 species have been identified as etiological agents of the disease. These protozoa are transmitted to animals and humans by insects of the Psycodidae family. Once infected, individuals can develop clinical symptoms such as prolonged fever and hepatosplenomegaly; if untreated, VL’s death rate can reach 95% [2]. The epidemiology of VL in Brazil in recent decades has shown a growing geospatial change. Previously restricted to rural habitats, new foci of transmission have been identified in urban areas, resulting from the adaptation of the vector insect to various environmental changes [3,4].

Visceral Leishmaniasis is a public health problem in Brazil, mainly in Maranhão, Piauí, Bahia, and Ceará, states of the Brazilian Northeast. Drought, common in the northeastern region of Brazil, has driven the rural exodus, and the presence of immigrants in the urban peripheries has been characterized as a source of infection for susceptible individuals. It is speculated that the intense migration of farmers from Piauí and Ceará in the 1980s, associated with the establishment of an industrial district and disorderly expansion of the urban environment, favored the expansion of VL in Maranhão [5,6,7].

According to the Brazilian Health Ministry Epidemiological Bulletin data on VL, Maranhão led the number of cases between 2012 and 2019, with 5308 cases and 377 deaths. Socioeconomic and environmental conditions and living habits may favor the spread of the disease [8]. Given the importance of VL in Maranhão territory, it becomes necessary to reflect on the main epidemiological aspects and distribution of the disease in the face of its substantial presence in this region.

## 2. Materials and Methods

**Figure 1:**
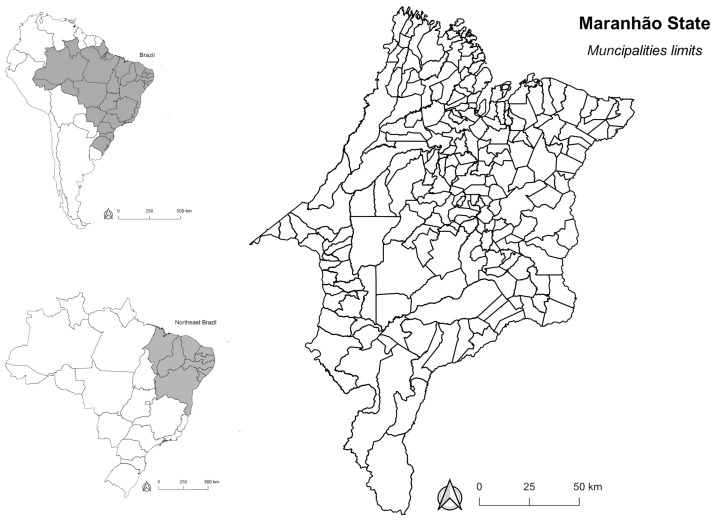
Geographical localization and municipality limits of Maranhão state.

This is a descriptive space–time and epidemiological study with a secondary data base that encompasses the cases of Visceral Leishmaniasis that occurred in the State of Maranhão between 2009 and 2020. The Maranhão state has 217 municipalities, 5 mesoregions, 21 microregions, and a demographic density of 19.81 inhabitants/km^2^. It has an HDI of 0.639 and is the 2nd poorest state in Brazil (Figure 1) [9].

The population consists of all cases of Visceral Leishmaniasis that occurred in the state of Maranhão from 2009 to 2020. The information was collected from the data base of the Brazilian Sistema de Informação de Agravos de Notificação (SINAN). The variables analyzed were age, sex, race/color, education, HIV coinfection, area of residence and confirmation criteria for VL. Data were organized and analyzed descriptively using Microsoft^®^ Office Excel 2010 software. Data were collected in the period 21–22 September 2023, and reviewed in the period 11–13 October 2023. The classification of VL transmission categories was made according to the following criteria: sporadic transmission—cities with an average of less than 2.4 cases; moderate transmission—cities with an average ≥ 2.4 and <4.4 cases; intense transmission—cities with an average ≥ 4.4 cases [10].

To calculate the incidence and prevalence rates, the resident populations according to IBGE projection for each year, available in the Datasus system, were used. For the elaboration of the maps, the data were tabulated in the Excel 2010 program and exported to the TabWin32 version 4.15 program for Windows.

Quantitative data were presented as mean and standard deviation. Qualitative data were presented by their absolute and relative frequencies. Time trend analysis was performed using the Jointpoint linear regression test (https://surveillance.cancer.gov/joinpoint/ accessed on 26 May 2023) to assess whether there was a trend of significant increase in the number of VL cases over the years, considering as the dependent variable the number of VL cases and as the independent variable the year in which the cases were recorded (2009–2020). The temporal trend was considered decreasing if both values of the 95% confidence interval (CI95) were negative; increasing, if these values were positive; and stationary when the confidence interval crossed the zero value, i.e., the lower and upper limits had opposite signs [11].

The spatial autocorrelation of VL prevalence was tested using the global Moran’s index, which ranges from −1 (negative autocorrelation) to +1 (positive autocorrelation), and 0 (zero) indicates the absence of spatial autocorrelation. Moran’s dispersion diagram was used to verify patterns of local association among states and their neighbors (Q1—municipalities with a high prevalence surrounded by municipalities that also have a high prevalence (High–High); Q2—municipalities with low prevalence surrounded by municipalities also with low prevalence (Low–Low); Q3—municipalities with high prevalence surrounded by states with low prevalence (High–Low); and Q4—states with low prevalence surrounded by states with high prevalence (Low–High)). Box and Moran maps were created to graphically visualize the spatial dependence of the data, considering the statistical significance and the association pattern. TerraView v. 5.3.6 software was used to analyze and calculate spatial autocorrelation indicators. Maps were created using QGIS v. 3.22.4 using publicly available shapefiles (available at www.ibge.gov.br, accessed on 15 April 2023). The chi square test was performed to assess differences between sexes using GraphPad Prism (v.8.0 for Windows program).

## 3. Results

From 2009 to 2020, 9210 cases of VL were notified in the Notifiable Diseases Information System of Brazilian Health Ministry, of which 5699 cases were confirmed, 2811 were discarded, and 700 cases were inconclusive. The mean of cases per year was 474.9 ± 157.2, corresponding to approximately 27.2% of the cases registered in the Northeast region and 14.7% of the cases in Brazil. The mean incidence was 6.5 cases/100,000 inhabitants and prevalence was 7.1 cases/100,000 inhabitants (Table 1).

**Figure 2:**
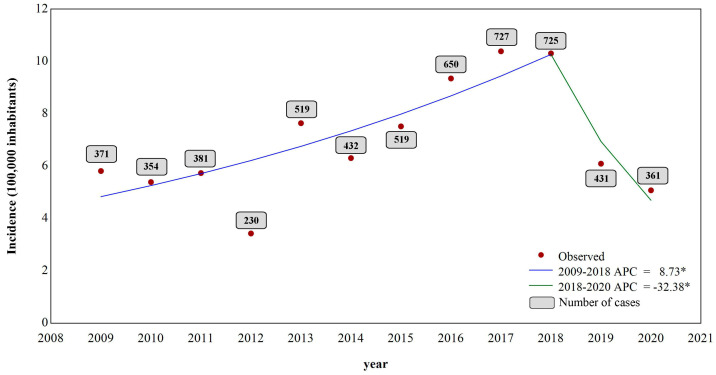
Jointpoint regression temporal analysis of the incidence and number of cases of VL from 2009 to 2020. * *p*-value ≤ 0.05.

The highest number of cases was notified in 2017, with 727 cases (12.8%), while 2012 registered 230 cases (4.0%). The jointpoint analysis of the VL incidence showed a significant growth between 2009 and 2018, with an annual change percentage (ACP) of 8.7% per year (*p* < 0.05 0.010; CI = 5.6; 16.1), despite the low number of cases registered in 2012. A change in the trend was observed in 2018, in which the second moment showed a significant decrease of 32.4% in the incidence (*p* < 0.05; CI = −49.5–−25.2) (Figure 2).

**Figure 3:**
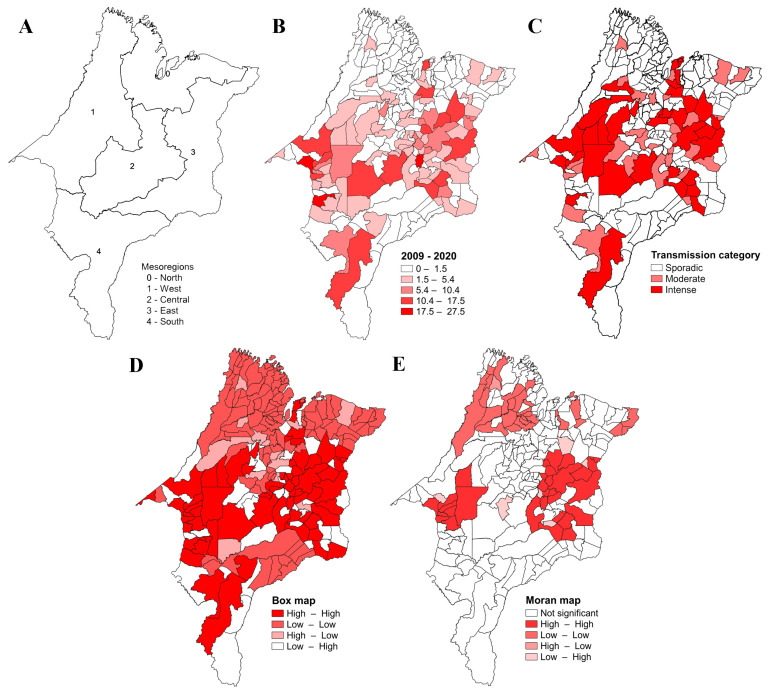
Geographical distribution and spatial analyses of the VL prevalence in Maranhão. Legend: (**A**) mesoregions of Maranhão; (**B**) mean accumulative prevalence of VL in Maranhão from 2009 to 2020; (**C**) category transmission of VL; (**D**) box map; (**E**) Moran map.

Regarding the geopolitical division of the five mesoregions of Maranhão, the North Maranhão mesoregion (60 municipalities; Figure 3A) had the highest number of cases (*n* = 2562 (45%)), followed by the mesoregions West Maranhão (52 municipalities) (*n* = 1209 (21.2%)), East Maranhão (44 municipalities) (*n* = 1041; (18.3%)), Center Maranhão (42 municipalities) (*n* = 636 (11.2%)), and South Maranhão (19 municipalities) (*n* = 251 (4.4%)) (Table 1).

All 21 Maranhão microregions reported cases of VL, corresponding to 204 (94%) of the 217 municipalities. The mean prevalence distribution from 2009 and 2020 is presented in Figure 3B. Approximately 31.7% of the cases in the state were registered in the urban agglomeration microregion of São Luís, which corresponds to about 40.8% of the cases in Maranhão, followed by the microregions of Imperatriz and Caxias. The highest incidence of the disease was registered in the microregions of Imperatriz (Western Maranhão), Presidente Dutra (Central Maranhão), and Urban Agglomeration of São Luís (Northern Maranhão), respectively; the highest prevalence was observed in the microregions of Imperatriz and Presidente Dutra, respectively (Table 1).

Regarding the transmission category, 35 of the 217 municipalities (16.1%) present intense transmission of VL, 32 (14.7%) municipalities present moderate transmission, and 150 (69.1%) municipalities present sporadic transmission (Figure 3C).

In the spatial analysis, the Moran’s Index showed a positive and significant result (I = 0.39; *p* = 0.001), indicating that VL’s prevalence is spatially dependent. The box map shows that 66 municipalities formed a Q1 cluster (High–High), 99 municipalities formed a Q2 cluster (Low–Low), 13 municipalities formed a Q3 cluster (High–Low), and 39 municipalities formed a Q4 cluster (Low–High). Q1-pattern clusters were mainly located in the Central Maranhão mesoregion, while Q2-pattern clusters are more evident in the North, East, West and South Maranhão mesoregions (Figure 3D). The Moran map shows two statistically significant areas in terms of spatial dependence with a Q1 pattern, one in the western mesoregion of Maranhão (9 municipalities) and another in the eastern mesoregion (22 municipalities). A third area with a Q2 pattern was identified in the Northern mesoregion (28 municipalities) (Figure 3E).

The sociodemographic assessment shows that 3642 (63.9%) cases were affected male (OR = 1.8; CI 95% = 1.72–1.92), and the average age was 16.8 ± 19.6 (range, 0–101) years, with the 1–4 age group (*n* = 1918 (33.7%)) being the most frequent. Approximately 74% (*n* = 4231) of the individuals considered themselves brown and only 4.5% (*n* = 254) of the individuals had completed elementary school, but this information was absent from the data for 61.7% (*n* = 3363) of the patients. When crossing age and education level variables, we observed that the majority of illiterate patients were over 19 years old (93.1% (Table 2).

Among the most reported symptoms were fever (*n* = 5318 (93.3%)), fatigue (*n* = 4703 (82.5%)), and edema (*n* = 2025 (35.5%)). It is noteworthy that around 25% of the patients had co-infections, with cases of VL concomitant with bacterial infections, such as leprosy and tuberculosis, and liver viruses. Co-infection with HIV was reported in 8.5% (*n* = 483) of cases. As for clinical progression, most patients evolved into a cure (*n* = 3743 (65.7%)), with pentavalent antimonials being the most used treatment (71.9%; *n* = 4098) (Table 3). VL led to the deaths of 6.8% (*n* = 386) of the patients, with a lethality rate of 6.9% and a mortality rate of 4.6% during the study period.

## 4. Discussion

Historically, the northeastern region of Brazil has been an endemic area for VL. Machado et al. [3] showed that the region features the highest number of cases in Brazil, with an average of 1973 cases per year between 2007 and 2017. Concerning Maranhão, our study revealed a high number of confirmed cases of VL, with records of the disease in all the state’s municipalities. The temporal analysis shows a point of change in the incidence of VL from 2018 onwards. This change in profile may be associated with the underreporting the disease due to the new coronavirus pandemic, which peaked in 2020. Thus, further studies are needed to assess whether the downward trend is persisting.

Analyses of the geographical distribution of the disease show that the North Maranhão mesoregion of the state had a higher prevalence of the disease. It can be explained by the concentration of cases registered in the municipality of São Luís, the state capital, which registered the highest number of cases (2251) between 2009 and 2020. Despite this, the spatial analysis showed no spatial dependence between São Luís and the neighboring municipalities.

A greater transmission activity and prevalence of VL in Maranhão seems to be concentrated in the more central region of the state. The spatial analysis confirms this finding, showing two hotspot areas with high values, one in the central-eastern Maranhão mesoregion and another in the central-western Maranhão region, and a hotspot area with low values can be seen in the northern mesoregion.

Our data show that VL mainly affects males, children under 14 years old, brown race, and those with incomplete elementary education. The disease can affect both sexes, but the prevalence of the disease in men can be explained by greater exposure to risk factors, such as phlebotomine vectors, and not by greater susceptibility [12]. However, some studies show that physiological factors are also a probable cause of the increased risk in men, indicating that after a certain age, sex hormones and the immune system in men result in greater susceptibility to infections and diseases [13]. Higher levels of testosterone have been associated with an increased risk of VL in India and Sudan [14,15]. These studies show a similar proportion to the VL cases in Maranhão. However, the study by Xavier-Gomes et al. [16] on 51 children from a municipality in Minas Gerais shows that 51% were female.

As for race, the majority were classified as brown, as reported in other surveys [17,18,19,20,21]. Researchers point out that susceptibility is universal, affecting people of all ages and sexes. However, a study on the epidemiology of VL in the state of Rio Grande do Norte found that black individuals with low levels of education were the most affected by the disease [17], while in the municipality of Bauru, São Paulo, 49.3% of reported cases of VL were of white race/skin color [21]. This difference is probably due to the different racial characteristics of the populations in the two municipalities.

As for schooling, as reported in studies carried out by Silva et al. [19] and Cavalcante et al. [22], the profile of notified VL cases was mainly represented by people with low levels of schooling, which is in line with the reported association between the occurrence of neglected infectious and parasitic diseases and populations with low levels of schooling [23]. Our results corroborate these previous studies, which show a low level of schooling, with the majority of patients being illiterate with incomplete primary education.

However, information on schooling was unavailable for 60.3% of the cases. These findings are similar to those described by Cavalcante [22] and Sousa [24], who also reported high percentages of data classified as “not applicable”. These results may be associated with the fact that patients under the age of 5 have not yet attended elementary school, and that this age group accounts for more than 50% of the VL cases.

Education, health, and income are the main elements when calculating a region’s human development index (HDI). In this scenario, education acts as a tool for health promotion, as it trains individuals in disease prevention methodologies, proper hygiene, and healthy nutrition [25].

Regarding age group, VL has a bimodal distribution, whereby it is possible to separate the population into two groups: children under the age of 10 and adults aged 20 to 59. The main group affected were children aged between 1 and 4 years old in over 32.8% of cases. Similar data were found in the study by Guerra [26] in Roraima between 1989 and 1993, with the higher number of cases in the 0 to 10 age group, which is typical of Visceral Leishmaniasis in the Americas. In the study by Scandar et al. [27], 45.4% of cases were concentrated in the 2- to 4-year age group, and in the study by Silva and Gaioso [28], in the 1- to 4-years group. More than 50% of patients in India, Sudan, and Brazil are children [29]. The greater susceptibility in children is explained by their relatively immature cellular immunity, aggravated by malnutrition, common in endemic areas, and greater exposure to the vector in the peridomicile [5].

Children, especially those under the age of five, are the age group most affected by the disease, a fact that may be associated with their more frequent contact with animals, but above all due to their humoral and cellular immaturity and the immunodepression induced by nutritional deficiency, a common situation in lower-income families. In addition, individuals aged between 20 and 39, considered economically active, are also highly susceptible to developing the disease [10,30].

VL showed a high cure rate (60.6%) and a low number (7.5%) of deaths when patients received adequate treatment for VL. This may be associated with improved techniques, access to diagnostic methods, and the high rate of patient treatment. VL diagnosis is based on clinical data, epidemiology, the microscopic visualization of the parasite in tissue aspirates (with variable sensitivity), serological tests (which have limitations), and polymerase chain reaction (PCR) [31,32]. In this study, laboratory criteria confirmed most cases. The introduction of immunochromatographic rapid tests into the public health system in Brazil was aimed at decentralizing the diagnosis of VL [33,34], which led to faster treatment. However, early diagnosis and treatment are not always achieved, and the most severe impact of late diagnosis and treatment is the death of the patient from VL [35]. Our data show that the Maranhão health system follows the Brazilian Ministry of Health treatment guidelines recommending pentavalent antimony (SbV) as the first-line therapy.

The WHO has been warning for decades that the emergence of HIV co-infection in Leishmaniasis-endemic areas is becoming a growing problem in developing countries and southern Europe [2]. In Maranhão, VL/HIV co-infection is similar to the national average (8.7%). The association between visceral leishmaniasis and HIV/AIDs can manifest as an aggressive disease or without specific symptoms, making diagnosis difficult [36]. HIV infection increases the risk of developing the disease in endemic areas by 100 to 2320 times, decreases the likelihood of a therapeutic response, and increases the risk of relapse. Concern about HIV–Leishmania co-infection is mainly related to the increased mortality rate [36,37].

As a limitation, our study is based on the analysis of secondary data in which several pieces of information are recorded as “ignored/blank”, which makes it difficult to provide a more reliable analysis of the impact of VL in Maranhão. According to Machado el al. [3], underreporting and the inadequate completion of information may have contributed a multifactorial cause in the processes of public health services.

## 5. Conclusion

VL continues to be an endemic problem in Maranhão; it is widely distributed throughout the study territory and mainly affects a socially vulnerable portion of the population. The study made it possible to identify priority areas and the epidemiological and clinical profile of the disease in the state, so that control measures can be developed. We also suggest that there be greater vigilance in the municipalities, as well as emphasizing the importance of all professionals filling out the forms completely, and that epidemiology centers be set up in the institutions and take on the task of reviewing the completeness of these data.

## Figures and Tables

**Table 1:** New cases, cases, incidence, and prevalence of Visceral Leishmaniasis distributed by geographic divisions in the period 2009 to 2020.

Mesoregion	Microregion (Municipalities)	Total Cases (%)	New Case	Case	Incidence	Prevalence
Center Maranhão	Alto Mearim e Grajaú (*n* = 11)	321 (5.6)	27.2 ± 17.0	28.2 ± 17.2	12.9 ± 6.8	12.9 ± 6.8
	Médio Mearim (*n* = 20)	126 (2.2)	9.2 ± 4.2	9.2 ± 4.2	6.2 ± 2.6	6.2 ± 2.6
	Presidente Dutra (*n* = 11)	189 (3.3)	15.9 ± 10.4	15.9 ± 10.4	16.2 ± 7.3	16.2 ± 7.3
East Maranhão	Coelho Neto (*n* = 4)	79 (1.4)	6.0 ± 2.1	6.0 ± 2.1	9.0 ± 2.6	9.0 ± 2.6
	Codó (*n* = 6)	280 (4.9)	23.2 ± 13.8	23.2 ± 13.8	9.0 ± 5.0	9.0 ± 5.0
	Chapadinha (*n* = 9)	145 (2.5)	12.5 ± 7.3	12.5 ± 7.9	12.5 ± 7.7	12.5 ± 7.7
	Baixo Parnaíba Maranhense (*n* = 6)	10 (0.2)	0.8 ± 0.6	0.8 ± 0.6	2.4 ± 1.7	2.4 ± 1.7
	Chapadas do Alto Itapecuru (*n* = 13)	107 (1.9)	8.1 ± 4.7	8.1 ± 4.7	8.6 ± 3.7	8.6 ± 3.7
	Caxias (*n* = 6)	420 (7.4)	32.0 ± 11.8	32.0 ± 11.8	10.2 ± 4.1	10.2 ± 4.0
North Maranhão	Baixada Maranhense (*n* = 21)	38 (0.7)	3.2 ± 3.6	3.4 ± 3.5	3.7± 2.7	3.7 ± 2.7
	Rosário (*n* = 8)	23 (0.4)	2.3 ± 2.1	2.3 ± 2.1	7.5 ± 7.8	7.5 ± 7.8
	Ocidental Maranhense (*n* = 13)	7 (0.1)	0.2 ± 0.4	0.2 ± 0.4	1.2 ± 2.5	1.2 ± 2.5
	Lençóis Maranhenses (*n* = 6)	29 (0.5)	2.1 ± 1.9	2.1 ± 1.9	2.5 ± 2.2	2.5 ± 2.2
	Itapecuru Mirim (*n* = 8)	142 (2.5)	7.6 ± 5.6	7.6 ± 5.6	7.3 ± 5.9	7.3 ± 5.9
	Aglomeração Urbana de São Luís (*n* = 4)	2323 (40.8)	184.7 ± 64.8	213.8 ± 81.7	14.3 ± 5.1	14.2 ± 5.1
West Maranhão	Imperatriz (*n* = 16)	986 (17.3)	74.3 ± 34.6	74.3 ± 34.1	16.7 ± 8.6	16.7 ± 8.6
	Pindaré (*n* = 22)	215 (3.8)	20.1 ± 12.5	20.1 ± 12.5	8.2 ± 3.9	8.2 ± 4.0
	Gurupi (*n* = 14)	8 (0.1)	0.7 ± 1.9	0.7 ± 1.9	2.0 ± 4.4	2.0 ± 4.4
South Maranhão	Gerais de Balsas (*n* = 5)	146 (2.6)	12.5 ± 5.4	12.5 ± 5.4	11.5 ± 4.6	11.5 ± 4.6
	Porto Franco (*n* = 6)	98 (1.7)	7.3 ± 6.4	7.3 ± 6.4	9.5 ± 7.6	9.5 ± 7.5
	Chapadas das Mangabeiras (*n* = 8)	7 (0.1)	0.5 ± 0.7	0.5 ± 0.7	4.3 ± 6.5	4.3 ± 6.5
Maranhão		5699	452.0 ± 141.5	497.5 ± 163.7	6.5 ± 2.0	7.1 ± 2.2
Northeast		22994	1741.7 ± 339.3	1921.0 ± 363.4	3.1 ± 0.6	3.1 ± 0.6
Brazil		42528	3169.5 ± 615.1	3493.0 ± 637.4	1.6 ± 0.3	1.6 ± 0.3

**Table 2:** Demographic profile of patients with Visceral Leishmaniasis in Maranhão state from 2009 to 2020.

Variables		
Sex	*n*	%
Female	2057	36.1
Male	3642	63.9
Age group (Years)		
<1	737	12.9
1–4	1918	33.7
5–9	543	9.5
10–14	242	4.2
15–19	214	3.8
20–39	1137	20.0
40–59	677	11.9
60–64	87	1.5
65–69	71	1.2
70–79	54	0.9
80+	19	0.3
Race/Skin color		
Ignored	109	1.9
White	571	10.0
Black	597	10.5
Yellow	64	1.1
Brown	4231	74.2
Indigenous	127	2.2
Level of Education		
Ignored/Blank	400	9.7
Illiterate	188	3.3
Incomplete 1st to 4th Elementary School	650	11.4
Complete 4th Elementary School	224	3.9
Incomplete 5th to 8th Elementary School	529	9.3
Complete primary education	150	2.6
Incomplete high school	161	2.8
Complete high school	254	4.5
Incomplete higher education	14	0.2
Complete higher education	16	0.3
Not applicable	2963	52.0

**Table 3:** Clinical profile of patients with Visceral Leishmaniasis in Maranhão state from 2009 to 2020.

**Symptoms**	***n* (%)**
Fever	5318 (93.3)
Fatigue	4703 (82.5)
Edema	2025 (35.5)
Weight loss	4463 (78.3)
Cough	2788 (48.9)
Pallor	4811 (84.4)
Splenomegaly	4847 (85.1)
Infectious condition	1424 (25.0)
Hemorrhage	540 (9.5)
Hepatomegaly	4071 (71.4)
Icterus	1800 (31.6)
Others	817 (14.3)
**Evolution**	***n* (%)**
Cure	3743 (65.7)
Abandonment	42 (0.7)
Death from VL	386 (6.8)
Death from other causes	114 (2.0)
Transfer	867 (15.2)
Ignored/Blank	547 (9.6)
**Treatment**	***n* (%)**
Pentavalent antimonial	4098 (71.9)
Amphotericin B lipoxomal	805 (14.1)
Pentamidine	9 (0.2)
Amphotericin B	283 (5.0)
Others	124 (2.2)
Not applied	90 (1.6)
Ignored/Blank	290 (5.1)

## Data Availability

All data used in this research were obtained from the Notifiable Diseases Information System of the Brazilian Ministry of Health available on the website https://datasus.saude.gov.br/acesso-a-informacao/doencas-e-agravos-de-notificacao-de-2007-em-diante-sinan/ accessed on 13 October 2023.
